# Ferroptosis as a Potential Cell Death Mechanism Against Cisplatin-Resistant Lung Cancer Cell Line

**DOI:** 10.34172/apb.2023.019

**Published:** 2021-11-10

**Authors:** Morteza Golbashirzadeh, Hamid Reza Heidari, Mehdi Talebi, Ahmad Yari Khosroushahi

**Affiliations:** ^1^Drug Applied Research Center, Tabriz University of Medical Sciences, Tabriz, Iran.; ^2^Department of Pharmaceutical Biotechnology, Faculty of Pharmacy, Tabriz University of Medical Sciences, Tabriz, Iran.; ^3^Hematology and Oncology Research Center, Department of Applied Cell Sciences, School of Advanced Medical Sciences, Tabriz University of Medical Sciences, Tabriz, Iran.; ^4^Department of Medical Nanotechnology, Faculty of Advanced Medical Science, Tabriz University of Medical Sciences, Tabriz, Iran.

**Keywords:** Drug resistance, Cisplatin, Apoptosis, Ferroptosis, Gene silencing

## Abstract

***Purpose:*** Drug resistance is a challenging issue in cancer chemotherapy. Cell death induction is one of the main strategies to overcome chemotherapy resistance. Notably, ferroptosis has been considered a critical cell death mechanism in recent years. Accordingly, in this study, the different cell death strategies focused on ferroptosis have been utilized to overcome cisplatin resistance in an *in vitro* lung cancer model.

***Methods:*** The physiological functions of Akt1 and GPX4, as critical targets for ferroptosis and apoptosis induction, were suppressed by siRNA or antagonistic agents in resistant A549 cells. Afterward, the interventions’ impacts on cell viability and reactive oxygen species (ROS) amount were analyzed by flow cytometry. Moreover, the alteration in the relevant gene and protein expression levels were quantified using Real-time PCR and western blot methods.

***Results:*** The result showed that the treatment with *Akt1* siRNA reversed the cisplatin resistance in the A549 cell line through the induction of apoptosis. Likewise, the combination treatment of the *GPX4* siRNA or FIN56 as ferroptosis inducers alongside cisplatin elevated ROS’s cellular level, reduced the cellular antioxidant genes level and increased the cisplatin cytotoxic effect.

***Conclusion:*** In conclusion, our study indicated that ferroptosis induction can be considered a promising cell death strategy in cisplatin-resistant cancer cells.

## Introduction

 Lung cancer is one of the fatal types of cancers worldwide.^1^ Non-small cell lung carcinoma (NSCLC) is the most common subtype of lung cancer with a high prevalence in the clinic.^1,2^ Based on Medscape (https://www.medscape.com), only 30% of NSCLC tumors have localized properties. Chemotherapy, instead of surgery/radiation therapy, is considered the first step of the treatment approach. Cisplatin is usually included as a first-line medicine in most NCLC chemotherapy protocols.^3-5^ Cisplatin exerts its effects by forming crosslinks in the DNA and inhibiting the DNA replication, G2/M phase cell cycle arrest, and inducing apoptosis.^6,7^ Nowadays, chemoresistance is the main reason for Cisplatin treatment failure.^8,9^ Galluzzi et al^10^ have classified cisplatin resistance mechanisms as pre-target resistance (e.g., drug efflux), on-target resistance (e.g., enhanced DNA repair machinery), post-target resistance (e.g., alternation in the drugs’ mechanism of action), and off-target resistance (alterations in compensatory signaling pathways); which were summarized in Table 1.

**Table 1:** Mechanism of Cisplatin resistance

**Cisplatin resistance**	**Mechanism of action**	**Reference**
Pre-target resistance	Avoiding to create cisplatin DNA adduct by decreasing cellular accumulation and efflux the cisplatin outside of the cell	^7,10,11^
On-target resistance	Enhanced DNA repair machinery and increase toleration of DNA	
Post-target resistance	Alternation or signaling pathway after DNA deficiency through cisplatin exposure	
Off-target resistance	An indirect cellular mechanism side effect that is not directly relative to cisplatin exposure but causes evasion of Apoptosis and Cisplatin cell death	

 Among various cellular signaling pathways, the PI3K-AKT has a higher impact on cancer progression and drug resistance. The PI3K-AKT regulates survival, differentiation, proliferation, migration, and chemoresistance in cancer cells. Consequently, targeting PI3K-AKT pathways has been considered a good strategy for battling cancer through Apoptosis induction.^12,13^

 However, as several other molecular pathways contribute to cancer survival and chemoresistance, overcoming the drug resistance for Apoptosis induction in different types of cancers might be challenging.^8,9,14^ So, opting for another cell death strategy might help re-sensitize these immortal cells.^15,16^

 Numerous types of cell death strategies have been implemented in cancer therapy research.^16-19^ Previous studies have remarkably revealed that reactive oxygen species (ROS) over-accumulation is a predominant phenomenon in cancer cells.^17-21^ Therefore, ROS-dependent, Caspase-independent programmed cell death, “ferroptosis,” attracted the researcher’s attention.^22,23^

 Particular criteria such as iron accumulation, lipid peroxidation, loss of mitochondrial function, and membrane integrity are ferroptosis hallmarks.^23^ Several compounds and siRNAs induce ferroptosis by interfering in the role of glutathione-peroxidase 4 (GPX4), Mevalonate pathway, cysteine-glutamate-anti-porters, and mitochondrial transporters.^24-26^ In this regard, FIN56, as one of the famous ferroptosis inducers, interacts with the squalene synthase enzyme in the Mevalonate-pathway, which leads to cytoplasmic CoQ10 depletion.^27,28^ Moreover, FIN56 indirectly promotes GPX4 degradation and causes cellular oxidants accumulation.^29,30^ The cellular CoQ10 depletion, besides higher amounts of cytoplasmic ROS, results in the peroxidation of cellular phospholipids, accumulation of the lipid ROS, and finally, ferroptosis induction.^31^

 Interestingly, ferroptosis induction by approved clinical anticancer drugs such as sulfasalazine, sorafenib, lapatinib, temozolomide, cisplatin,^21^ and even by cytotoxic T-cells^26^ have been reported.

 Considerable researches indicate that ferroptosis inducers would attain a good percentage of the novel cancer therapeutics,^32,33^ especially in chemotherapy resistance forms. Favorably, Roh et al have reported that ferroptosis induction through silencing specific genes such as cystine-glutamate-antiporter (xCT) can increase cisplatin’s efficacy in cisplatin-resistant cancer cells.^16^ Similarly, according to Sugiyama, xCT inhibitor sulfasalazine eradicates paclitaxel-resistant uterine serous carcinoma.^34^ Likewise, GPX4 siRNA was used for ferroptosis induction in chemoresistance aggressive Panc-1 cancer stem-like cells.^26^

 Despite these achievements, ferroptosis’s effectiveness vs. apoptosis in eradicating cancer resistance cells was not addressed. Therefore, this study tested the efficacy of these two cell death strategies combined with Cisplatin drug in the cisplatin-resistant A549 as the NSCLC model. At first, ferroptosis effectiveness was examined using GPX4 siRNA and FIN56 agents; then, apoptosis efficacy was investigated with *AKT1* siRNA. Finally, the ability of these coadministrations in eradicating resistance A549 cells was compared.

## Materials and Methods

###  Cell culture 

 The cisplatin-resistant lung cancer cell line (A549 CDDP) was generously gifted by Dr. Roya Salehi, faculty of Advanced Medical Science, Tabriz University of Medical Science, Tabriz, Iran. The normal human foreskin fibroblasts (HFFs) cell line was purchased from the National Cell Bank Pasteur institute of Iran. The cell lines were cultured in RPMI-1640 medium (Gibco, MD, USA) supplemented with 10% fetal bovine serum (FBS) (Gibco, MD, USA) and (penicillin 100 U/mL and streptomycin 100 µg/mL) (Inoclon Co, Iran, 12PS2-100) at 37°C, humidified 5% CO_2_.

###  Cell death induction and Cytotoxicity assay

 To assess the possibility of using the ferroptosis strategy in combating cancerous resistance cells, we applied both chemical (FIN56) and biological (GPX4 siRNA) treatments. Moreover, Akt1 siRNA was used to evaluate the effect of the Apoptosis induction strategy in this battle.

 MTT test was performed to determine the effective dose of FIN56 against the resistant A549 and HFF normal cell lines. In brief, the resistant A549 and HFF cell lines were seeded with the cell density of 1 × 10^4^ cells/well in 96-well microplates and were treated with different FIN56 concentrations (0, 5, 10, 12, 14, 18, 20, 22, 25, 30, 35 μM) for 48 hours, and subsequently subjected to the MTT assay as previously described.^35^

 The GPX4 and Akt1 siRNAs (Table 2) were designed by the siRNA direct website (http://design.RNAi.jp/); and purchased from Eurofins Genomics Company (Ebersberg, Germany). The siRNA transfection procedure was conducted as in the previous study.^36^ In brief: the resistant A549 cell line was seeded in a 6-well plate one day before transfection at an initial density of 0.7 × 10^5^ cells/well. Then, based on the numerous previous published papers,^37-39^ 100 nM of each siRNAs were complexed with HiPerFect reagent (Qiagen, Germany) in a serum-free media, and mixtures were applied to the cells. After one hour of incubation, treatment media was removed, and the cells were washed with PBS and finally further incubated in the complete media for 48 hours.^40^

**Table 2:** The sequence of siRNA

**Gene**	**siRNA sequence **	**Length**
AKT-1	S: 5′- CCAUGAACGAGUUUGAGUACC -3′	21 nt
	A: 5′- UACUCAAACUCGUUCAUGGUC -3′	
GPX4	S: 5′- CUACAACGUCAAAUUCGAUAU -3′	21 nt
	A: 5′- AUCGAAUUUGACGUUGUAGCC -3′	

S: Sense, A: Anti-sense.

###  Ferroptosis assay

 Cellular lipid ROS and total ROS were assessed after treating cells with FIN56 and GPX4 siRNA by DCFDA and Boron dipyrromethene (BODIPY) dyes according to published protocols^41^; The emitted fluorescence was detected by a FACSCalibur flow-cytometry (Becton Dickinson, USA).

###  Apoptosis assay 

 Determination and analysis of Apoptosis after treatment by *Akt1* siRNA and cisplatin has been performed by Annexin V FITC/PI test flowed by published protocol.^42^

###  Western blot analysis

 The Akt1 and GPX4 proteins’ expression was measured by western blot analysis after siRNA transfection followed by the published protocol.^43^ In a few words, proteins were lysed for 10 minutes on ice after extraction. Then, the extracted proteins were separated by 12.5% SDS-PAGE and transferred to a polyvinylidene difluoride membrane blocked by BSA. The blocked membrane was incubated with the desired primary antibodies. Finally, horseradish peroxidase-conjugated secondary antibodies were applied with ECL reagent to the reaction based on the manufacturer’s instructions.

###  RNA isolation, cDNA synthesis, and real-time PCR

 Real-time PCR was performed for determining the expression of *AKT1, GPX4, Nerf2,* and *CoQ10* after treatment by FIN56 and the siRNAs in relation to GAPDH as the internal control. Resistance A549 cell line was seeded in a 6-well plate and treated as mentioned in the previous section. Total cellular RNA isolation and cDNA synthesis were performed based on our previously published paper.^36^ According to the manufacturer’s instructions, the total RNA was extracted by Triazole (GeneAll Biotech, South Korea) reagent. The amount and purity of total RNA were measured by Nanodrop 260/280 nm (Thermo Scientific^TM^ NanoDrop). To synthesize cDNA, 1 µg of total mRNA was used based on a commercially available protocol of BioFact cDNA synthesis kit (Daejeon, South Korea).^44^ Amplification and alternation of target genes were performed by StepOne^TM^ Real-Time PCR System instrument (Applied Biosystems, USA) with SYBR Green detection system. Suitable primers were designed by NCBI primer blast (https://www.ncbi.nlm.nih.gov/tools/primer-blast), as mentioned in Table 3. The amplification reaction was performed in a 20 μL final volume which contained 1 μL cDNA sample, 2 μL F and R primers (20 pmol), 10 μL Master-mix, and 7 μL RNAs free water. The PCR program was carried out for 40 cycles: first denaturation time at 95°C for 20 minutes, which is followed by 40 cycles of 95°C for 20 seconds, an ideal annealing temperature (Table 3) for 30 seconds; and 72°C for 10 seconds. Afterward, to acquire melting curves, the temperature increased step by step from 65°C to 95°C. Finally, the relative expression of genes was calculated using the ∆∆ Ct method.

**Table 3:** List of primers used for detecting specific RNAs using real-time PCR

**Gene**	**Sequence**	**Annealing**	**Fragment length**
*GAPDH*	F: 5′-TTGACCTCAACTACAGGTTTACA -3′ R: 5′-GCTCCTCCTGGAAGATGGTGATG -3′	60°C	100
GPX4	F: 5′-TGGGAAATGCCATCAAGTGGA-3′ R: 5′-GGGCAGGTCCTTCTCTATCAC-3′	60°C	123
*AKT1*	F: 5′-GAGTTCACGGCCCAGATGAT-3′ R: 5′-CGAGTAGGAGAACTGGGGGA-3′	57°C	105
*Nrf2*	F: 5′-ATGCCACAGGACATTGAGCA-3′ R: 5′-TTGGCTTCTGGACTTGGAAC-3′	60°C	119
*CoQ10B*	F: 5′-TTGGATTTCCACCTGTGTTG-3′ R: 5′-CGCCAAATAGTCTCCAAATGA-3′	59°C	118

F: forward primer, R: reverse primer.

###  Trypan blue exclusion and Cell viability assay

 The Trypan blue exclusion assay was conducted to evaluate the viability of cell lines after treatments. The resistant A549 cell line was seeded in a 6-well plate. Cells separately were treated with FIN56 (5μM), the Akt1 siRNA (100 nM), or the GPX4 siRNA (100 nM), along with cisplatin (1μM) for 48 hours to set up a combination effect. Then, cells were trypsinized and incubated with Trypan Blue solution (0.4% Trypan Blue, Merck, Germany) for 10 minutes. The percentage of viable and dead cells was measured by FACSCalibur flow-cytometry (Becton Dickinson, USA).

## Results and Discussion

###  Ferroptosis and apoptosis against chemotherapy-resistance lung cancer cells

 Several genetically or epigenetically alterations in cancer cells, like gene rearrangements, pathogenic gene mutations, gene expression, post-transcriptional and translational regulation by non-coding RNAs, are responsible for the heterogeneity of different cancers.^45-52^ These heterogeneities make it impossible to provide a unique magic bullet for cancer treatment. Meanwhile, due to cancer cells’ ever-changing nature, the inherited or acquired resistance forms of cancer cells accumulated in the tumor cells’ environment, which led to a relapse of resistant tumors.^53,54^

 Considering the clinical data of NSCLC in the NCBI ClinVar database, the primary oncogenic driver activating mutations frequently occur in *EGFR*, *HER2, MET*, *BRAF*, *RAS*, *PIK3CA*, and *MAP2K1* genes. Moreover, chromosomal instabilities, translocation, and fusion of oncogenes such asanaplastic lymphoma kinase (ALK) greatly impact cancer cell generation.^13,54,55^

 Several genetic expression alterations and mutations establish inherited or acquired resistance NSCLC by altering cancer-related proteins’ activity or therapeutic target site (on target resistance). For example, ERCC1 and RRM1 repairing proteins’ overexpression has been associated with gemcitabine and cisplatin resistance tumors. The mutated *EGFR, BRAF, ALK, KRAS *cancer cells are resistant to various tyrosine kinase inhibitors (TKIs)^56-59^; and, losing the transmembrane domain of PD-L1 in some mRNA splicing variants led to resistance to anti-PD-1 treatment.^60^

 Moreover, overexpression of the *EGFR*, *c-MET*, *HER2*, *FGFR3*, and *AXL* TKIs serves as compensatory signaling pathways such as PI3K-Akt, RAS-ERK, STAT establish off-target resistance to the TKI’s in NSCLC patients.^61-65^

 Additionally, the role of long and small non-coding RNAs in NSCLC chemotherapy resistance is undeniable. Mainly LncRNAs, through alternating the expression of drug efflux proteins, apoptosis, and autophagy modulating proteins, can establish the on-target resistance in NSCLC tumors.^66^ Besides the roles mentioned above, LncRNAs by induction of cancer stem-cell-like phenotypes and Epithelial-mesenchymal transition can augment the compensatory signaling pathways and establish the off-target resistance in NSCLC.^67^

 The A549 cell line, isolated from human lung alveolar epithelial cell carcinoma, is considered one of the standard NSCLC models for *in vitro* chemotherapy studies.^68^ Different cancer cell sub-populations with various genetic mutations, phenotypes, and sensitivity to chemotherapeutic drugs are present in this heterogeneous lung cancer model.^69-71^

**Figure 1:**
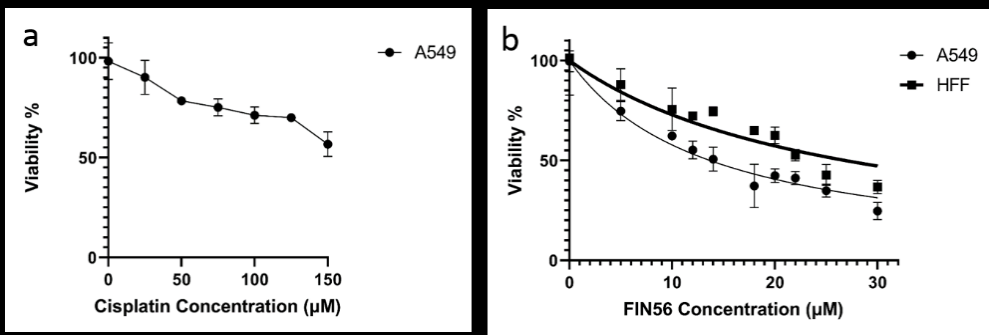
Cisplatin-resistant A549 cells’ sensitivity to cisplatin (a) and FIN56 (b) were evaluated by MTT assay. Cancerous A549 has higher sensitivity to FIN56 than normal HFF cells.

 The clinically relevant cisplatin concentration is about 14 µM in the patient’s plasma,^72^ which is remarkably lower than the expected IC50 of 252.7 µM in the A549 resistance cell line (Figure 1a). Since applying more than 14 µM of cisplatin to the normal cell is highly toxic, in this study, a combination of chemotherapy and gene therapy strategies was applied to assess the possibility of eradicating the cisplatin-resistant A549 cell line, using ferroptosis or apoptosis induction.

 Ferroptosis is a ROS-dependent and caspase-independent cell death pathway, which is naturally applied by cytotoxic killer cells to eradicate tumor cells.^73,74^ In ferroptosis, usually cellular lipid peroxidation levels are augmented due to the accumulation of ROS molecules. The excessive cellular ROS production in ferroptosis is related to the malfunction of mitochondrial membrane potential, cellular thiol-dependent antioxidant system malfunction, and cellular antioxidant regulatory pathways such as mevalonate and Nrf2 pathways.^75,76^

 Notably, ROS plays diverse roles in cancer cell’s fate. Based on the cancer cells’ distinct metabolism and tumor hypoxia, the cancer cells have higher ROS concentrations than normal cells. The increased ROS level can induce DNA mutagenesis and help the heterogenicity of the tumors. Moreover, ROS molecules’ continuous exposure can persuade cell proliferation by activating oncogenic proteins, including growth factor receptors, VEGF, Ras, MAPK, and PI3K/AKT. Furthermore, ROS molecules elevate the activity of Nrf2, FOXO, and HIF1α antioxidant transcription factors and subsequent antioxidant enzymes in cancer cells. This procedure led to a higher level of the cellular antioxidant system such as Heme oxygenase, glutathione peroxidase (GPX), superoxide dismutase, catalase, and glutathione (GSH) in cancer cells.^77^

 However, as most chemotherapeutic drugs are ROS generators, these compounds’ administration can alter the cancer cells’ redox homeostasis. The elevated ROS concentrations suppress regular cellular enzymatic activity and cell connectivity, cause DNA damage, and cease cell cycle progression. ROS, while at higher concentrations, impairing the cells’ physiological function, results in a distinct type of regulated cell death such as ferroptosis.^78^

 Therefore, ROS-dependent cell death strategies such as ferroptosis have been used in cancer therapy, especially for eradicating the resistant forms of cancers.^77,79^

###  FIN56 and GPX4 siRNA induce ferroptosis in A549 cisplatin-resistant cells

 In this study, two ferroptosis inducers, FIN56 small molecule and, GPX4-siRNA were harnessed in the battle against cisplatin-resistant A549 cells. We used the MTT assay to determine the cytotoxicity of FIN56 in both cancerous and normal cell lines. As shown in (Figure 1b), both cell lines’ viability was reduced after 48 hours incubation with FIN56 in a dose-dependent manner. However, the IC50 value of FIN56 for A549 and HFF was calculated as 12.71 and 24.97 μM, respectively. Therefore, these results indicate that resistant A549 cells are more sensitive to FIN56 than the HFF normal cells. Additionally, as 5µM of the FIN56 is sufficient for inducing the ferroptosis, and the normal cells have an upper than 80% viability (Figure 1b), this concentration opted for the rest of this study.

**Figure 2:**
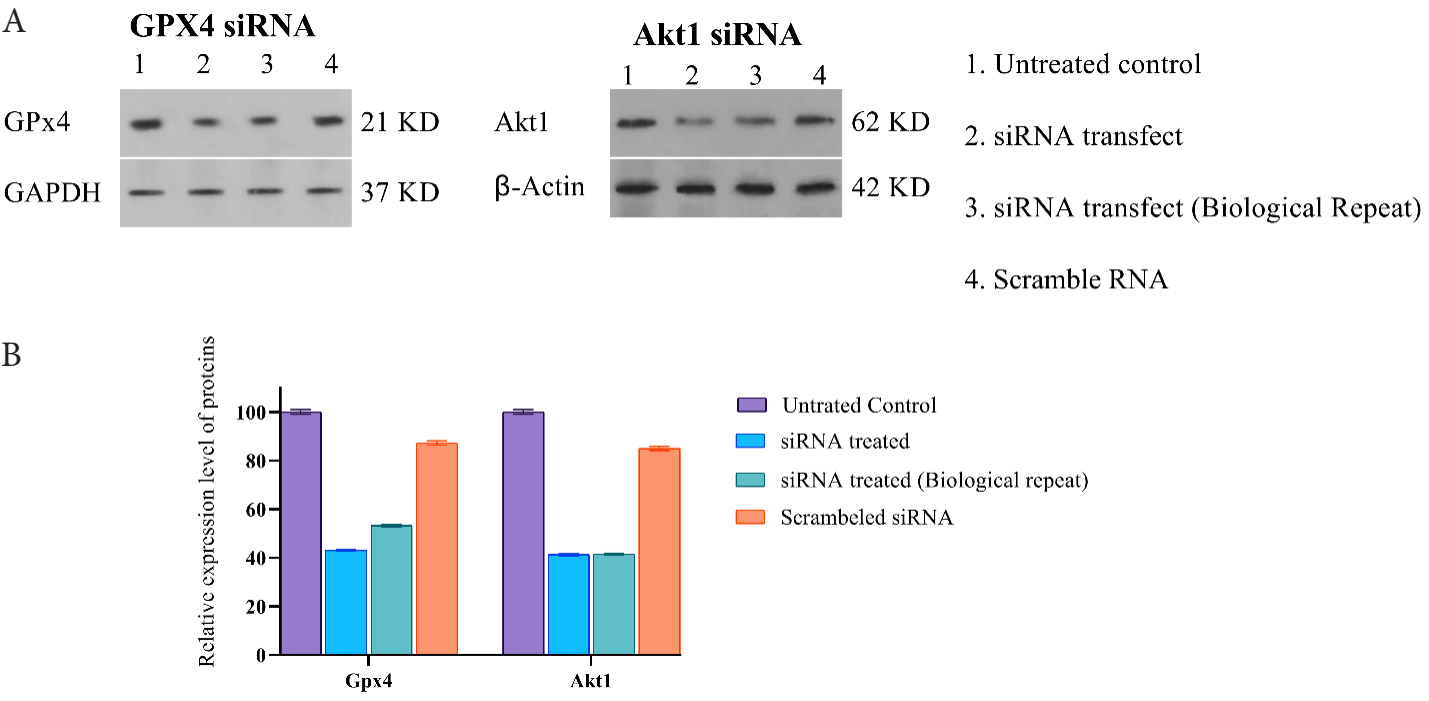
Effects of Akt1 and GPX4 siRNA on the expression of GPX4 and AKT1 in A549 cells. siRNAs were added to the A549 cell culture for 48 h, and cells were harvested to be processed for Western blot analysis. (A) Protein bands were shown as above, Lane 1: control; lanes 2&3: 1ng Akt1 siRNA; lane 4: scramble RNA. (B) Viability of A549 cell line after treatment by siRNAs for 48h

 Along with the chemical induction of ferroptosis, *GPX4* siRNA gene silencing was also performed. To determine the efficacy of gene silencing, we performed the western blot analysis after 48 hours of siRNA incubation. We observed that GPX4 protein’s quantity decreased by 35%, calculated using ImageJ software, compared to their control groups, as shown in (Figure 2).

**Figure 3:**
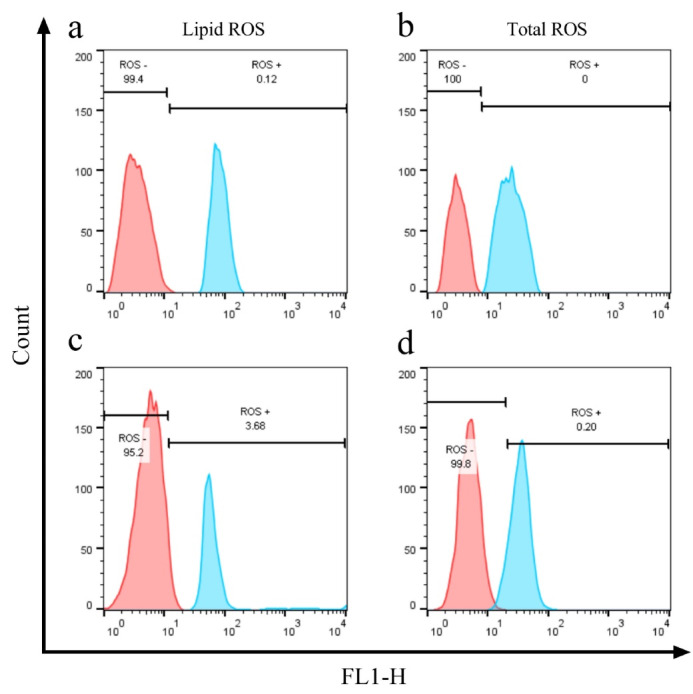
Effect of FIN 56 (Panels a and b) and GPX4 siRNA (Panels c and d) on the cellular lipid and total ROS levels. Cells were treated with 5μM FIN56 and 1 ng siRNA for 48 h. DCFDA and BODIPY-C11, respectively, measured total ROS and lipid ROS levels

 The cellular accumulation of ROS molecules is considered hallmarks of ferroptosis induction. The emission of fluorescent dyes DCFDA and BODIPY-C11 was assessed by flow-cytometry to confirm the ferroptosis induction in FIN56 and *GPX4* siRNA-treated A549 cells. As shown in (Figure 3), the total and lipid ROS amount were shifted to higher signals in FIN56 (5μM), and *GPX4* siRNA (100nM) treated cells (48 hours) compared to their control groups, which indicates successful ferroptosis induction in both treatments.

###  Akt1 siRNA induce ferroptosis in A549 cisplatin-resistant cells

 There are numerous reports on the role of the Akt and its cooperator proteins such as PI3K, mTOR, NF-κB, c-Met, c-Myc, and ERK1/2 in cisplatin resistance induction in the A549 cell line.^80-83^ These signaling pathways alter apoptotic (Bax, Bad, Bim) and anti-apoptotic (Bcl2, Bcl-xl) gene expression levels, inhibiting apoptosis induction in these resistance cells. Therefore, Akt-related pathways have been the center of attention in several chemoresistances re-sensitization studies.^84-88^

 Based on previous *in vitro* reports, administration of vinorelbine, sunitinib, BAICALEIN, or genistein alleviates the cytotoxicity of cisplatin in the resistance of the A549 cell line through inhibition of the Akt pathway and other cisplatin resistance-related mechanisms such as drug metabolism, efflux, and DNA repair machinery.^52,89-91^

 Correspondingly, gene therapy strategies also have been applied to overcome chemoresistance in cisplatin-based therapies. Replacement gene therapy of tumor suppressor genes such as PTEN,^92^ IL-24^93,94^ re-sensitize the cisplatin-resistant A549 cells via downregulation of the PI3K/AKT/hTERT pathway. Similarly, knocking down the overexpressed aldehyde dehydrogenase 1A1,^95^ Tripartite motif-containing 59 oncogene protein,^96^ and MDR1^97^ restore the cisplatin toxicity in the resistance A549 cell line in an Akt dependent manner.

 Similarly, in this study, silencing the *Akt1* gene expression using the 100 nM, specific siRNA was opted to eradicate the cisplatin-resistant A549 cells. The western blot analysis after 48 hours of siRNA incubation indicates that total amounts of Akt1 protein were successfully downregulated distinctively to 41% (Figure 2).

**Figure 4:**
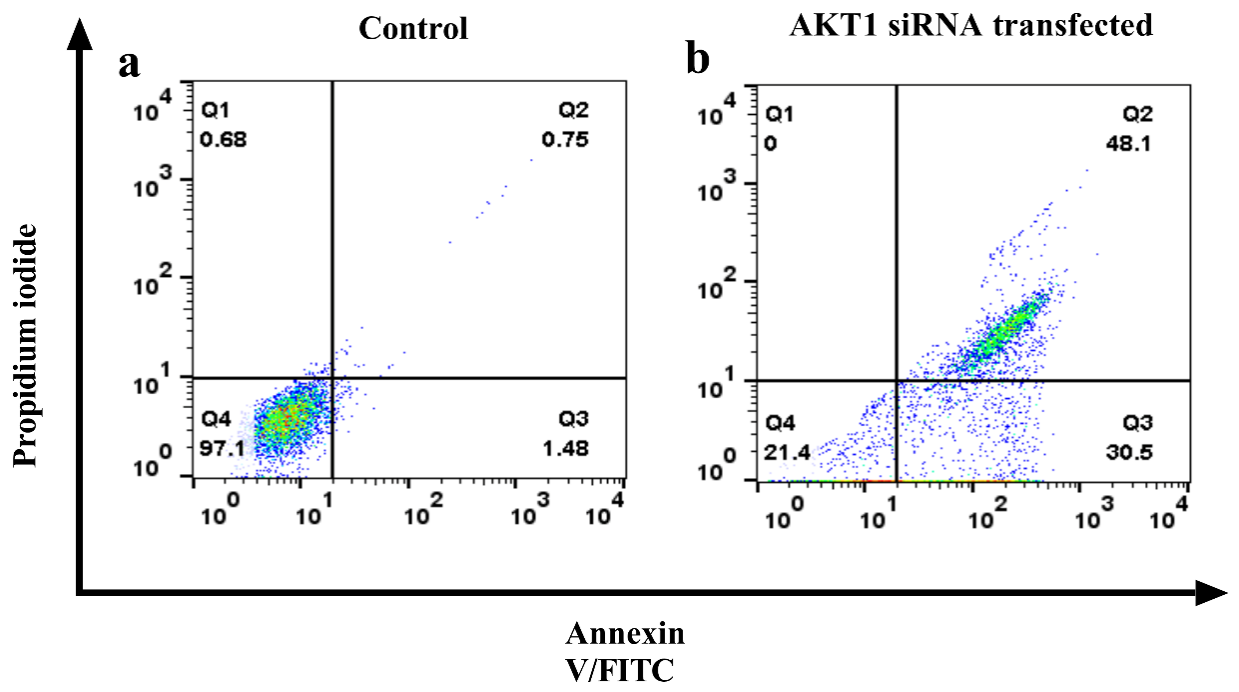
Flow-cytometry analysis of treated Akt1 siRNA and the control group. The panels are shown in the following order: (a) control group; (b) Akt1 siRNA treated group. Dots with Annexin V−/PI + (Q1), Annexin V + /PI + (Q2), Annexin V + /PI− (Q3), and Annexin V−/PI− (Q4) and features represent necrotically, late apoptotic, early apoptotic, and viable intact cells, respectively

 Correspondingly, the flow-cytometry technique’s shift of Annexin V/PI stained cells revealed that *Akt1* siRNA increased the percentage of late apoptotic cells from 0.75% to 48.1% and early apoptotic cells from 1.48% to 30.5% meaningfully (Figure 4). Therefore, the *Akt1* siRNA effectively induces apoptosis in the A549 resistant cells.

###  Antioxidant related genes were down-regulated after FIN56 and siRNA treatments

 Based on the previous knowledge about ferroptosis and its inducers, the ROS accumulation in the cells is negatively correlated with the level and activity of the antioxidant gene regulator (*NRF2* and *AhR*), antioxidant enzymes (*GPX4*), and antioxidant molecules (*CoQ10*, glutathione).^73,98,99^ Mainly, ferroptosis inducers disturb the function of this antioxidant redox balance regulator system.^100^ However, these antioxidant systems’ elevated levels and activity can induce resistance against ferroptosis in a cell-type-specific manner.^101,102^

**Figure 5:**
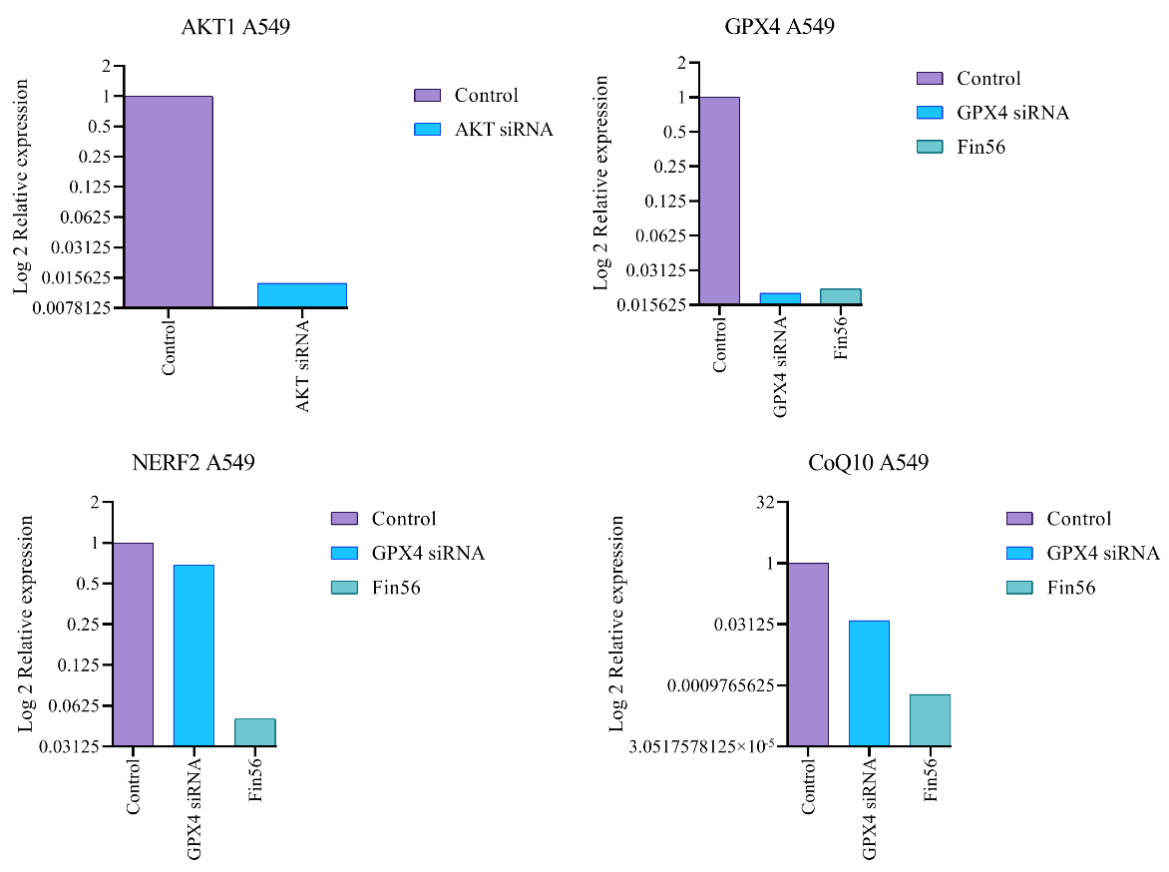
Akt1, GPX4, CoQ10, and, Nerf2 gene expression ratios were obtained by real-time PCR with treatments (Akt1 and GPX4 siRNAs separately and FIN56) in resistant A549 cell lines. Target genes were normalized to GAPDH as a control gene

 Correspondingly, this study evaluated the expression variation of anti-ferroptosis and anti-apoptosis-related genes, mainly *GPX4, CoQ10, Nerf2*, and *Akt1* genes, using a real-time PCR technique. The graphical representation of different treatments’ gene expression ratios presents in (Figure 5). Considering the ferroptosis induction, after the treatment of FIN56, the gene expression ratio of *GPX4, CoQ10, and Nerf2* was calculated as 0.0216, 0.00059, and 0.05 compared to the non-treated control group. While following the transfection of *GPX4* siRNA, the gene expression ratio of the mentioned genes was 0.02, 0.039, and 0.69, respectively. The results reveal that following either *GPX4*-siRNA or FIN56 treatments, the expression level of anti-ferroptosis *Nrf2*, *GPX4*, and *CoQ10* genes was significantly declined (*P* < 0.001) compared to the correspondence untreated control group. Likewise, for apoptosis induction, the *Akt1* gene expression ratio in *Akt1* siRNA-treated A549 cells was 0.0138, indicating effective inhibition of the *Akt1* expression.

###  FIN56, along with GPX4 and Akt1 siRNAs, destroy A549 cisplatin-resistant cells

**Figure 6:**
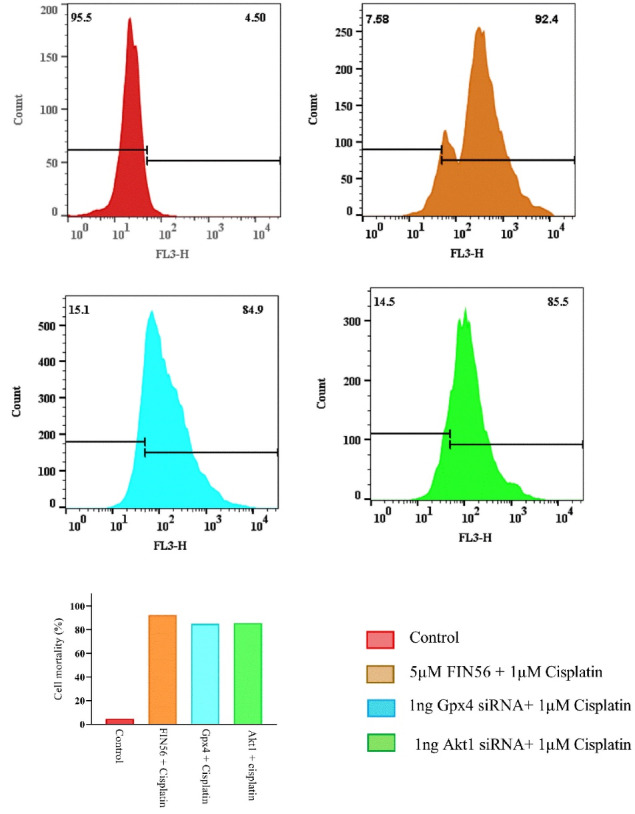
Histogram analysis of the trypan blue exclusion assay by flow-cytometry to evaluate cells’ mortality after treating by 5μM FIN56 + Cisplatin, 1 ng Akt1 siRNA + Cisplatin, 1 ng Gpx4 siRNA + cisplatin compared with the control group

 The results of this indicated cell viability alterations in combination therapy of “Cisplatin combined by ferroptosis or apoptosis inducers” in A549 resistant cells by Trypan blue exclusion dye assay, using the Flow cytometry technique. As shown in (Figure 6), the highest percentage of dead cells was observed after treatment with “Cisplatin + 5μM FIN56” at 92%. Meanwhile, administration of either “Cisplatin + 100 ng *Gpx4* siRNA” or “Cisplatin + 100 ng *Akt1* siRNA” results in 85% dead cells compared to the control group.

 Similarly, several reports from Roh and colleagues highlight the ferroptosis cell death strategy’s impact on eradicating the cisplatin-resistant cancer models.^16,103^ They showed that Erastin and Sulfasalazine could be used to overcome cisplatin resistance through ferroptosis induction. However, another ferroptosis inducer, like RSL3 activity against the same cisplatin-resistant cell line, depends on the NRF2 pathway activity.^15^ Similarly, RNA sequencing data of the Erastin sensitive and resistant cell lines reveals that the transcriptional activity of NRF2 and AhR is one of the most critical factors in the ferroptosis-related resistance phenotype. Principally, it was shown that A549 cells, as an epithelial lung cancer model, have NRF2 and AhR mediated resistance to Erastin. However, A549 transdifferentiated mesenchymal lung cancer cells, which have chemoresistance to some therapeutics, are sensitive to erastin.^104^

 This difference in the A549 ferroptosis sensitivity may also be related to the mechanism of ferroptosis inducers. Erastin and Sulfasalazine are Cystine-Glutamate-antiporter inhibitors that lead to cellular glutathione depletion. However, RSL3 is a GPX4 antagonist, and FIN56, like siRNA, depletes cellular GPX4 protein, leading to lipid ROS accumulation.^21^

 Previous studies show that directly targeting the Akt using siRNA can induce apoptosis in resistance cells.^105,106^ Similarly, in this study, based on the Trypan blue exclusion assay and Annexin-PI staining flow cytometry results, Akt1 downregulation using the siRNA induced about 80% programed apoptosis death. This outcome might be related to the down-regulation of the Akt1-dependent several anti-apoptotic proteins such as the Bad and Bcl families.^107^

## Conclusion

 In conclusion, chemotherapy resistance NSCLC cells could be eradicated either by reinforcing the apoptosis by targeting the Akt1 as a critical cellular survival regulator; or by disrupting the cellular ROS homeostasis using ferroptosis inducers. Therefore, Akt1 or GPX4 siRNA combined with drug administration could be considered a promising strategy in NSCLC therapy.

## Acknowledgments

 This project is part of a Ph.D. thesis (grant No. 59305) funded by Drug Applied Research Center, Faculty of Pharmacy, Tabriz University of Medical Sciences, Tabriz, Iran.

## Author Contributions

**Conceptualization: **Ahmad Yari Khosroushahi.

**Data curation: **Morteza Golbashirzadeh.

**Formal Analysis:** Morteza Golbashirzadeh.

**Funding acquisition: **Hamid Reza Heidari.

**Investigation: **Morteza Golbashirzadeh.

**Methodology: **Ahmad Yari Khosroushahi,, Mehdi Talebi.

**Project administration: **Hamid Reza Heidari.

**Resources: **Ahmad Yari Khosroushahi.

**Supervision: A**hmad Yari Khosroushahi.

**Validation: **Hamid Reza Heidari.

**Visualization: **Mehdi Talebi.

**Writing – original draft: **Morteza Golbashirzadeh.

**Writing – review & editing: **Hamid Reza Heidari.

## Ethical Issues

 Not applicable.

## Conflict of Interest

 The authors declare no conflict of interest.
